# Normatização de programas de acesso expandido e uso compassivo de medicamentos na América do Sul

**DOI:** 10.26633/RPSP.2019.57

**Published:** 2019-07-08

**Authors:** Gabriela Bittencourt Gonzalez Mosegui, Fernando Antoñanzas

**Affiliations:** 1 Universidade Federal Fluminense, Instituto de Saúde Coletiva, Departamento de Saúde e Sociedade Universidade Federal Fluminense, Instituto de Saúde Coletiva, Departamento de Saúde e Sociedade Niterói (RJ) Brazil Universidade Federal Fluminense, Instituto de Saúde Coletiva, Departamento de Saúde e Sociedade, Niterói (RJ) Brasil.; 2 Universidad de La Rioja, Departamento de Economia e Empresa Universidad de La Rioja, Departamento de Economia e Empresa La Rioja Espanha Universidad de La Rioja, Departamento de Economia e Empresa, La Rioja, Espanha.

**Keywords:** Ensaios de uso compassivo, registro de produtos, legislação sanitária, América do Sul, Compassionate use trials, products registration, health legislation, South America, Ensayos de uso compasivo, registro de productos, legislación sanitaria, América del Sur

## Abstract

**Objetivo.:**

Descrever e comparar os marcos regulatórios das políticas de acesso rápido/alternativo a medicamentos (acesso expandido e uso compassivo) em países da América do Sul.

**Métodos.:**

Realizou-se um estudo exploratório e descritivo, com análise documental. Além de revisão da literatura científica sobre o tema, foram levantadas informações e normas oficiais caso estivessem disponíveis nas páginas eletrônicas das autoridades reguladoras de medicamentos. Foram coletadas informações sobre a forma como cada país define os conceitos de acesso expandido e uso compassivo, fase clínica em que o medicamento fica disponível para esses usos alternativos e obrigações de médicos e patrocinadores.

**Resultados.:**

A partir dos critérios de inclusão, foram selecionados para o estudo Argentina, Brasil, Chile, Peru e Uruguai. O levantamento de informações mostrou que Argentina e Brasil apresentam um cenário regulatório mais estruturado. O Chile apresenta uma norma sobre acesso expandido e uso compassivo, porém sem definir explicitamente esses conceitos. No Peru e Uruguai, foi constatada a ausência de definições importantes quanto ao acesso expandido. Não foram identificadas quaisquer bases de dados com informações sobre acesso expandido e uso compassivo, corroborando a percepção de escassez de dados empíricos para avaliar os resultados dessas políticas.

**Conclusões.:**

Todos os países analisados contam com um marco regulatório que permite o acesso rápido/alternativo a medicamentos por pacientes em situação de risco. Porém, não existem bancos de dados e transparência de informações que permitam caracterizar quais medicamentos e pacientes se beneficiam desse acesso alternativo e avaliar os resultados dessas políticas na América do Sul.

## Abstract (en)

**Objective.:**

To describe and compare the regulatory framework governing policies on rapid/alternative access to medicines (expanded access and compassionate use) in South American countries.

**Method.:**

An exploratory descriptive study with analysis of documents was performed. In addition to a literature review, official rules issued by regulatory agencies were reviewed if available. Information was collected on how countries define the concepts of expanded access and compassionate use, clinical phase in which the medicine becomes available for these alternative uses, and role of physicians and sponsors.

**Results.:**

Argentina, Brazil, Chile, Peru, and Uruguay were included in the study. The information obtained revealed that the regulatory scenario is more structured in Argentina and Brazil than in the other countries. In Chile, rules on expanded access and compassionate use are available, however without an explicit definition of these concepts. In Peru and Uruguay, important definitions are missing regarding expanded access. The search did not reveal any databases with information on expanded access and compassionate use programs, supporting the notion that empirical data to evaluate the results of these policies are lacking.

**Conclusions.:**

All the countries analyzed have a regulatory framework that contemplates rapid/alternative access to medicines by patients at risk. However, databases and transparent information are lacking, preventing a snapshot of the medicines covered and patients benefiting from alternative access programs and assessments of these policies in South America.

## RESUMEN

**Objetivo.:**

Describir y comparar los marcos regulatorios de las políticas de acceso rápido y alternativo a medicamentos (acceso ampliado y uso compasivo) en países de América del Sur.

**Métodos.:**

Se realizó un estudio exploratorio y descriptivo, con análisis documental. Además de revisión de la bibliografía científica sobre el tema, se obtuvo información sobre las normas oficiales, si estaba disponible en las páginas electrónicas de las autoridades regulatorias de medicamentos. Se recolectó información sobre la forma en que cada país define los conceptos de acceso ampliado y uso compasivo, la fase clínica en que se ofrece el medicamento para uso alternativo y las obligaciones de los médicos y patrocinadores.

**Resultados.:**

A partir de los criterios de inclusión, se seleccionaron para el estudio Argentina, Brasil, Chile, Perú y Uruguay. La información obtenida mostró que Argentina y Brasil presentan un escenario regulatorio más estructurado en comparación con los demás países del grupo citado. Chile presenta una norma sobre acceso ampliado y uso compasivo, pero sin definir explícitamente esos conceptos. En Perú y Uruguay, se constató la falta de definiciones importantes en cuanto al acceso ampliado. No se encontraron bases de datos con información sobre acceso ampliado y uso compasivo, lo cual corroboró la percepción sobre la escasez de datos empíricos para evaluar los resultados de esas políticas.

**Conclusiones.:**

Todos los países analizados cuentan con un marco regulatorio que permite el acceso rápido y alternativo a medicamentos de los pacientes en situación de riesgo. Sin embargo, no existen bancos de datos ni información transparente que permitan determinar qué medicamentos y pacientes se benefician de ese acceso alternativo y evaluar los resultados de esas políticas en América del Sur.

Todo novo medicamento, para ser registrado e comercializado, deve cumprir um complexo e por vezes demorado procedimento regulatório, que pode levar de 6 a 18 meses (1, 2). Garante-se, desse modo, que, quando utilizado pelo paciente, o medicamento será seguro, eficaz, de qualidade e claramente indicado para o tratamento em questão.

O acesso rápido, alternativo ao acesso convencional, pode ser definido como qualquer política que torne mais flexível a disponibilidade de medicamentos a um paciente ou grupo de pacientes, diminuindo o período de espera e autorizando legalmente o uso. Nos países desenvolvidos, os sistemas sanitários e a indústria farmacêutica vêm trabalhando há décadas para acelerar o acesso a novos medicamentos indicados para situações-limite, especialmente quando não há outra alternativa disponível (1). Em 2015, o registro de novos medicamentos exigia 478 dias na Europa e 304 dias nos Estados Unidos (2).

Nos Estados Unidos, a agência reguladora *Food and Drug Administration* (FDA) desenvolveu quatro abordagens distintas, conhecidas de forma genérica como revisão acelerada de medicamentos, para disponibilizá-los o mais rapidamente possível: análise prioritária, terapia inovadora, aprovação acelerada e revisão rápida (3). Medicamentos enquadrados na revisão prioritária podem ser aprovados em até 6 meses, contra 10 meses previstos no registro padrão. O processo de terapia inovadora agiliza o desenvolvimento e revisão de medicamentos que possam demonstrar melhoria substancial em relação à terapia já disponível. A aprovação acelerada permite que medicamentos para doenças graves, que preenchem uma necessidade médica não atendida, possam ser aprovados com base em um desfecho substituto. Finalmente, a revisão rápida foi projetada para facilitar o desenvolvimento e agilizar a liberação de medicamentos que preencham uma necessidade médica não atendida no tratamento de doenças graves.

Na União Europeia, a Agência Europeia de Medicamentos (EMA) é a principal responsável pelo acesso rápido aos medicamentos. Assim como os países membros em suas regulações nacionais, tem poder para autorizar e legislar outras vias de acesso. Para fortalecer o desenvolvimento de medicamentos inovadores, que oferecessem grande vantagem terapêutica em relação aos tratamentos existentes ou beneficiassem pacientes sem opções de tratamento, a EMA propôs o sistema *PRIority MEdicines Scheme* (PRIME). Os medicamentos contemplados no PRIME, considerados prioritários pela EMA do ponto de vista da saúde pública, devem-se mostrar promissores para necessidades médicas não satisfeitas. O esquema fundamenta-se na interação e diálogo prévios com a indústria farmacêutica, otimizando os planos de desenvolvimento e flexibilizando as rígidas normas sanitárias de registro e acelerando o acesso a medicamentos que possam ter impacto na qualidade de vida de pacientes com enfermidades graves e debilitantes (4, 5).

Antoñanzas et al. (6) resumem três abordagens do PRIME usadas no processo regulatório: avaliação acelerada (em especial para inovações terapêuticas de interesse para a saúde pública), autorização condicional para comercialização (contempla medicamentos dirigidos a necessidades médicas não atendidas, como medicamentos órfãos para doenças raras) e uso compassivo, com definição semelhante à empregada pela FDA (utilização de medicamentos ainda não aprovados para registro, em pacientes individuais, com doenças debilitantes, para as quais não há tratamentos eficazes). Essas abordagens aumentariam a possibilidade de diálogo entre os sistemas de saúde e a indústria farmacêutica, que direcionaria suas pesquisas a áreas de interesse público, ao mesmo tempo alcançando mais precocemente o mercado. Além disso, as abordagens de acesso rápido aos medicamentos podem servir como vias alternativas para os sistemas de saúde e pacientes – para os sistemas de saúde, oferecendo a chance de apoiar pacientes ou grupos de pacientes que não teriam opções de tratamento; para os pacientes portadores de doenças debilitantes graves ou que ameaçam a vida, sem proposta terapêutica satisfatória disponível, acesso a medicamentos ainda sem registro. A indústria sempre reconheceu esses programas como parte de uma política de investimento em pesquisa e desenvolvimento. Ao comercializarem drogas experimentais, podem antecipar a recuperação do custo da pesquisa por meio dos lucros obtidos com essa comercialização, patentes e fidelização do prescritor (7).

A América Latina representa um mercado potencial para a comercialização de medicamentos. Diversos países latino-americanos possuem sistemas de regulação de preços que, apesar de estabelecerem limites, adotam regras que estimulam a concorrência no setor (6, 8, 9). Entretanto, não há evidências que comprovem que as normativas adotadas pelas agências sanitárias sul-americanas são suficientes para facilitar o acesso a medicamentos prioritários; além disso, não se sabe até que ponto a avaliação de resultados dessas políticas está prevista nos marcos legais. Nesse contexto, os objetivos deste trabalho foram descrever e comparar os marcos regulatórios de uso compassivo e acesso expandido a medicamentos em países da América do Sul e identificar bases de dados sobre o tema, caracterizando os medicamentos, pacientes beneficiados e o alcance dessas políticas.

## MATERIAIS E MÉTODOS

Realizou-se um estudo exploratório e descritivo. Na fase exploratória, foram feitas buscas e análises da literatura científica sobre o tema. Com vistas a investigar a viabilidade de acesso a dados secundários, exploraram-se as páginas eletrônicas das instituições de países da América do Sul responsáveis pela regulação de medicamentos. A partir dessa análise, definiram-se como critérios de inclusão a disponibilidade de um conjunto mínimo de informações institucionais, estruturais e funcionais, de informações sobre os processos de regulação de medicamentos e informações sobre acesso acelerado e uso compassivo, disponíveis em páginas eletrônicas institucionais das autoridades reguladoras de países da América do Sul. Foram selecionadas as seguintes agências: Agência Nacional de Vigilância Sanitária (ANVISA), Brasil; *Administración Nacional de Medicamentos, Alimentos y Tecnologías* (ANMAT), Argentina; *Instituto de Salud Publica del Chile* (ISP), Chile; *Instituto Nacional de Salud* (INS), Peru; e *Ministerio de Salud*, Uruguai.

Em uma segunda fase, compararam-se as normas e o panorama de regulação de uso compassivo, assim como os programas de acesso expandido a medicamentos, nos países selecionados. Como fontes de dados foram utilizadas informações e normas oficiais disponíveis nas páginas eletrônicas dessas autoridades reguladoras. Também foram consultadas as páginas oficiais para averiguação de dados que caracterizassem o perfil dos medicamentos empregados, dos pacientes e dos custos dos programas.

Após a coleta, os dados foram agrupados segundo as seguintes categorias: definição do país para os conceitos de acesso expandido e uso compassivo, fase clínica em que o medicamento torna-se disponível e obrigações de médicos e patrocinadores. Os resultados foram comparados descritivamente entre as instituições/países. Artigos de revistas especializadas e relatórios que continham informações importantes para o desenvolvimento do estudo, bem como documentos, legislação, dissertações e teses foram consultados para embasar a discussão.

## RESULTADOS

O levantamento e análise das principais normas relacionadas ao acesso rápido a medicamentos em vigor na Argentina, Brasil, Chile, Peru e Uruguai identificou a existência de abordagens normativas ao uso compassivo de medicamentos em todos os países. Foram identificadas abordagens normativas de acesso expandido na Argentina, Chile e Brasil, onde também existe abordagem para fornecimento de medicamento pós-estudos. Os demais países não mencionam o fornecimento de medicamento pós-estudos em suas normas sanitárias, mas citam a possibilidade de pacientes obterem esses insumos (10-15). Uma recente norma publicada pela ANVISA criou o registro acelerado de medicamentos no Brasil (16). As principais características dos programas de acesso expandido e uso compassivo de medicamentos nos países estudados são apresentadas na tabela 1.

**TABELA 1.** Principais características dos programas de acesso expandido e uso compassivo em países selecionados da América Latina

País/variável	Acesso expandido	Uso compassivo
Argentina		
Definição	Programa para pacientes com alto risco de morte ou severa deterioração da qualidade de vida para os quais não existe tratamento eficaz disponível no país – normativa vigente – ano 2017 (18)	Regime de Acesso de Exceção para paciente sem alternativa terapêutica adequada e cujas condições clínicas sejam contraindicadas para uso de medicamentos disponíveis no país – normativa vigente – ano 2016 (17)
Fase clínica em que o medicamento fica disponível	Medicamentos com fase II já finalizada	Medicamento em qualquer fase de pesquisa clínica
Obrigações de médicos e patrocinadores	Patrocinador fornece tratamento e assistência; médico trata o paciente Programa de Acesso Expandido (PAE) presta assistência médica	Médico presta assistência médica
Brasil		
Definição	Programa para grupo de pacientes sem alternativa terapêutica satisfatória – normativa vigente – ano 2013 (20)	Programa para uso individual de paciente sem alternativa terapêutica satisfatória – normativa vigente – 2013
Fase clínica em que medicamento fica disponível	No mínimo fase III de pesquisa clínica	Medicamento em qualquer fase de pesquisa clínica
Obrigações de médicos e patrocinadores	Médico presta assistência médica; patrocinador fornece tratamento e assistência ao evento adverso	Médico presta assistência médica; patrocinador fornece o tratamento
Chile		
Definição	Pacientes sem alternativas terapêuticas comparáveis ou satisfatórias, quando o médico responsável considere indispensável a utilização de um medicamento sem aprovação sanitária vigente, poderão fazê-lo – normativa vigente – ano 2013 (14)	“Situação de uso compassivo” sem definição nas normas sanitárias; normativa vigente – ano 2013 (14)
Fase clínica em que medicamento fica disponível	Sem informação	Sem informação
Obrigações de médicos e patrocinadores	Patrocinador fornece tratamento e assistência ao evento adverso. Investigador deve garantir segurança e bem-estar dos participantes durante a pesquisa e, no protocolo, devem estar claros os resguardos necessários aos eventos adversos	Patrocinador fornece tratamento e assistência ao evento adverso. Investigador deve garantir segurança e bem-estar dos participantes durante a pesquisa e, no protocolo, devem estar claros e os resguardos necessários aos eventos adversos
Peru		
Definição	Sem informação	Programa para uso individual de paciente sem alternativa terapêutica satisfatória – normativa vigente – ano 2016 (10)
Fase clínica em que medicamento fica disponível	Sem informação	Produto em investigação ou medicamento conhecido empregando dose e vias de administração diferentes das estabelecidas
Obrigações de médicos e patrocinadores	Sem informação	Médico presta assistência para reação adversa a medicamentos
Uruguai		
Definição	Sem informação	Programa para uso individual de paciente sem alternativa terapêutica satisfatória – normativa vigente – ano 2013 (24)
Fase clínica em que medicamento fica disponível	Sem informação	Produto em investigação ou medicamento conhecido empregando dose e vias de administração diferentes das estabelecidas
Obrigações de médicos e patrocinadores	Sem informação	Médico presta assistência em caso de reação adversa a medicamentos; a correspondente também tem responsabilidade

## Argentina

Na Argentina, as disposições da ANMAT tratam do direito de acesso a medicamentos inovadores ainda não disponíveis no mercado. A disposição 10 401/16 (17) regulamenta um regime de acesso excepcional, que anteriormente chamava-se uso compassivo de medicamentos. Nessa disposição, o Regime de Acesso de Exceção a Medicamentos (RAEM) é definido, estabelecendo-se um procedimento para a entrada de medicamentos destinados ao tratamento de um paciente individual para o qual não exista alterativa terapêutica adequada no país. Esses medicamentos devem ser comercializados no seu país de origem e, caso estejam em fase de pesquisa clínica, deverão estar registrados pela autoridade sanitária do país onde a pesquisa esteja sendo realizada (17-19).

Na disposição 828/17 (18), a Argentina estabelece um Programa de Acesso Expandido (PAE) destinado a grupos de pacientes com alto risco de morte ou deterioração da qualidade de vida, que exijam tratamento com medicamentos ainda não comercializados no país, de forma gratuita (18). Também nesse país há definição da fase em que o estudo clínico deve encontrar-se para que o paciente solicite este acesso (tabela 1).

### Brasil

No Brasil, a ANVISA normatizou o direito de acesso a medicamentos inovadores que ainda não estão disponíveis no mercado. A medida alcança pacientes portadores de doenças debilitantes e graves para as quais não há medicação ou para as quais é insuficiente o tratamento disponível (20, 21). Foram regulamentados três programas que podem beneficiar pacientes nessa condição: uso compassivo, acesso expandido e fornecimento de medicamento pós-estudos. O primeiro trata de uma autorização emitida pela ANVISA para que a indústria execute determinado programa assistencial no país, fornecendo medicamento novo, promissor e ainda sem registro. O programa possibilita que a empresa seja autorizada a importar medicamentos não registrados no país para tratar doenças raras e graves. O acesso expandido ocorre quando se disponibiliza um medicamento novo, promissor, ainda sem registro na ANVISA ou não disponível comercialmente no país, que esteja em estudo clínico em fase III, em desenvolvimento ou concluído.

Uma recente norma publicada pela ANVISA criou o registro acelerado de medicamentos no Brasil. A RDC 204 determina critérios para enquadramento na categoria prioritária dos pedidos de registro, de pós-registro e de anuência prévia em pesquisa clínica de medicamentos (16). A obtenção do registro ocorre em até 120 dias. O registro acelerado pode ser conseguido para medicamentos empregados no tratamento de doenças negligenciadas, medicamentos novos e vacinas ou soros hiperimunes (16). Após a obtenção e publicação do registro do medicamento, inicia-se o prazo de 30 dias para solicitação de preço máximo, nos termos regulados pela Câmara de Regulação do Mercado de Medicamentos (CMED).

### Chile

É atribuição do ISP, organismo do Estado, outorgar autorização para comercialização e distribuição de medicamentos, mediante registro sanitário. As resoluções 57/2001 e 108/2013 (13, 14), que tratam dos requisitos a serem cumpridos pelos estudos clínicos com agentes farmacológicos em seres humanos e aprova o instrutivo de farmacovigilância, respectivamente, citam apenas “situação de uso compassivo” de medicamentos no Chile (13, 14). Os pacientes podem também obter medicamentos sem registro sanitário por meio de uma solicitação de “importação de uso provisório”. Essa importação é feita individualmente por pacientes com doenças graves ou condição que ameace iminentemente suas vidas, para as quais não há alternativas terapêuticas comparáveis ou satisfatórias. O pedido é realizado por um médico que considere essencial o uso de um medicamento sem aprovação sanitária (22). A norma técnica 57 confere poderes ao diretor do ISP para autorizar o uso de medicamentos sem registro sanitário prévio. Entretanto, essa norma técnica não abrange uso de produtos já autorizados para um uso diferente do registrado (*off label*), ou para uso por outra via de administração.

Assim, a legislação chilena sobre acesso expandido ou uso compassivo não é explícita.

### Peru

No Peru, o uso excepcional de medicamentos é tratado de forma acessória e pontual na regulamentação nacional de ensaios clínicos controlados (ECC) através dos decretos supremos 017-2006-SA e 021-2017-SA, aprovados em 2006 e 2017, respectivamente, pela Oficina Geral de Investigação e Transferência Tecnológica (OGITT), vinculada ao Instituto Nacional de Saúde, órgão de regulamentação sanitária do país (10, 12). O regulamento de 2006 possui dois artigos que tratam da definição e dos requisitos para o uso compassivo (tabela 1).

### Uruguai

O Uruguai comercializa somente medicamentos e produtos médicos importados que estejam registrados. Excepcionalmente, o Ministério da Saúde pode autorizar um paciente, com uma prescrição específica, sob responsabilidade de um médico, a importar um produto não registrado, quando não haja similar terapêutico circulando no mercado uruguaio (23). A instrução 13 221-008, de 2013, faz menção ao uso compassivo em regulamento de Ensaios Clínicos Controlados do Mercosul de 1996 (24), ao citar estudos de fase I, que devem ser realizados em voluntários sãos, com algumas exceções. Tais exceções talvez sejam pacientes doentes, em uso compassivo de medicamentos. Há uma nota de 2013 na legislação uruguaia (23) que menciona o uso estendido na definição de farmacovigilância, retirada de um documento do Mercosul (24).

## DISCUSSÃO

Existem políticas de apoio ao desenvolvimento de medicamentos para necessidades médicas não atendidas e que aceleram o acesso a esses insumos em países da União Europeia e nos Estados Unidos, entre outros (1-6). Entretanto, na América do Sul, apesar de uma resolução referente ao tema no Brasil, não há outros avanços nesse caminho (16). As normativas dos países analisados incluem aspectos relacionados ao acesso expandido a ao uso compassivo, mas distanciam-se das abordagens usadas pela FDA (análise prioritária, terapia inovadora, aprovação acelerada e revisão rápida) ou EMA (avaliação acelerada e autorização condicional para comercialização) (1, 3-6)*.* Essas abordagens aumentariam a possibilidade de diálogo entre a indústria farmacêutica e os sistemas de saúde, no sentido de direcionar pesquisas a áreas de interesse, e poderiam servir como alternativas para sistemas de saúde e pacientes.

Para a condução dos programas de acesso expandido e uso compassivo é necessário que sejam cumpridas as determinações sobre o acesso prioritário, e o tempo de obtenção do registro é importante. Mais relevante será o programa de acesso expandido quanto maior for o tempo exigido para o processo padrão de registro de medicamentos no país. Contudo, a agilidade não deve significar ausência de evidências de eficácia, nem a sustentação em evidências não científicas, que levariam ao insucesso clínico ou a reações adversas. Os processos de análise de registro de medicamentos por revisão acelerada, acesso expandido e uso compassivo devem ser rigorosos (25-27). Por outro lado, são escassos os dados publicados sobre como o mecanismo de aprovação acelerada diminui o prazo de disponibilidade de novas tecnologias em comparação ao processo tradicional (2).

Na Argentina, data de 1995 a norma que regula as solicitações pessoais de uso compassivo de medicamentos. Definições mais claras encontram-se em disposições legais mais recentes, de 2016 e 2017 (17, 19). O Brasil possui um arcabouço legal um pouco mais organizado sobre o tema desde 2013; mas desde 1976 já permitia o uso de medicamentos experimentais em pacientes que não pudessem participar de ECC (28). As abordagens encontradas nos demais países indicam, prioritariamente, normatizações acerca do uso compassivo, datando de 2001 no Peru, 2011 no Chile e 2013 no Uruguai (13, 15, 23). Promulgado em 1992 pela FDA e em 2007 pela EMA, o uso compassivo tem caráter complexo, com elementos semelhantes, porém natureza diferente daquela dos ensaios clínicos (1, 3, 4, 29).

As definições de uso compassivo empregadas nos países sul-americanos pesquisados são semelhantes às usadas por EMA e FDA (14). A Argentina passou a utilizar a denominação Regime de Acesso de Exceção a Medicamentos em substituição ao termo uso compassivo; a disposição onde se encontra o termo está relacionada a importação de produtos sem registro (17, 18). O mesmo acontece no Chile, onde, apesar dos regulamentos relacionados à condução de ECC citarem apenas “situação de uso compassivo”, há um instrutivo de importação de produtos sem registro sanitário para condições que ameacem a vida, para as quais não existem alternativas terapêuticas satisfatórias (13, 14, 22). A norma peruana relacionada à condução de ensaios clínicos define o uso compassivo (13). O Uruguai, assim como os demais países analisados, possui um instrutivo de importação de medicamentos sem registro, por meio de solicitação médica premente. Supõem-se que esses medicamentos sejam de uso compassivo, mas não foi identificada nenhuma norma onde o termo apareça (23).

Estados Unidos e Europa lidam com programas de acesso expandido da mesma forma, mas empregando distintas terminologias. A FDA chama de acesso expandido o uso de qualquer dispositivo sob investigação para tratamento de um paciente fora de um ECC (1). Se o paciente, ou grupo de pacientes, não atender os critérios de inclusão estabelecidos para o ensaio, mas tiver potencial para receber o medicamento sob pesquisa para uma condição para a qual não exista terapia adequada estabelecida, pode ser um candidato ao acesso expandido. A EMA não possui um termo para acesso expandido; emprega “uso compassivo” a um grupo de pacientes, ao invés de um indivíduo prioritariamente, como fizeram alguns países sul-americanos (4, 5).

Na prática, ainda não se sabe como o acesso expandido e o uso compassivo se aplicam nos países da América do Sul, dada a escassez de dados empíricos. Os poucos achados para o Brasil sobre autorização para uso de medicamentos em acesso expandido e uso compassivo mostram diferenças entre os registros encontrados no repositório *Clinical Trials* (www.clinicaltrials.gov) e os registros com denominação “uso compassivo” ou “acesso expandido” encontrados no Portal de ensaios clínicos autorizados no país (http://portal.anvisa.gov.br/consulta-de-ensaios-clinicos-autorizados). Os relatórios da Coordenação de Pesquisa Clínica em Medicamentos e Produtos Biológicos (COPEC) de 2016 e 2017 apenas tangenciam o assunto (30-32). Para traçar e avaliar políticas e programas de acesso rápido, é importante que existam informações consolidadas e que possam ser consultadas.

No contexto da disponibilidade de dados, Kim et al. (27) verificaram a frequência com que medicamentos para câncer são aprovados com base em desfechos substitutos e se estudos posteriores apontam uma melhora da sobrevivência global de pacientes em uso dessas tecnologias. Compararam-se medicamentos que adotaram o caminho tradicional *versus* o acelerado, aprovados pela FDA, ao longo de 5 anos. Os autores concluíram que apenas um medicamento do grupo acelerado, contra quatro do caminho tradicional, mostrou desfechos significativos após 5 anos de seguimento. Esse exercício gerou debates sobre os resultados em saúde das políticas de acesso rápido, quanto ao benefício aos pacientes e também quanto à penetração mais rápida da indústria farmacêutica no mercado.

Algumas limitações deste trabalho devem ser apontadas. Nem todos os países sul-americanos foram contemplados – porém os países investigados correspondem a aproximadamente 75% da população sul-americana (33). Consultaram-se bases oficiais, contendo documentos de governo e de gestão das autoridades reguladoras nacionais, mas não é possível assegurar que todos os dados sejam absolutamente corretos e todas as normas tenham sido incluídas. Foi possível perceber discrepâncias entre as informações divulgadas nos *sites* das agências, a legislação correspondente e os dados das buscas da revisão de literatura. Por fim, embora o estudo tenha procurado tratar de forma equânime as cinco autoridades reguladoras estudadas, há maior quantidade de literatura e informações disponíveis a respeito da regulação de medicamentos no Brasil e Argentina.

Nos Estados Unidos, na Europa e em outros países do mundo, o acesso rápido está na agenda política sanitária como uma via de avanço, incentivando o diálogo precoce entre as autoridades sanitárias e a indústria farmacêutica, com agilização burocrática e do registro de patentes, negociação de preços, discussão de mecanismos de reembolso e criação de diretrizes e protocolos terapêuticos para acesso a medicamentos, sendo os cidadãos os reais beneficiários. O diálogo entre empresas e agências e a transparência e fácil acesso a dados podem impulsionar essas políticas e demonstrar seu alcance.

A diversidade nas normas e a ausência de definições importantes e de clareza dificultaram a análise de alguns países da América do Sul. Os programas de uso compassivo e acesso expandido podem ser a única maneira de pacientes com doenças graves e raras acessarem um novo medicamento. Os países da América do Sul têm ainda um longo caminho regulatório a percorrer. De qualquer forma, um primeiro passo foi dado em relação às legislações de enfermidades raras, onde uma avaliação acelerada é normatizada desde 2008 e 2012 no Brasil e na Argentina respectivamente. Os exemplos de FDA e EMA indicam que a flexibilidade regulatória pode estar associada a cuidado e qualidade no acesso. Entretanto, são necessárias informações que permitam a caracterização dos medicamentos, pacientes e custos envolvidos e dados que facilitem a análise do alcance desses programas.

## Contribuição dos autores.

CBGM e FA elaboraram o projeto, coletaram e revisaram os dados, interpretaram os resultados e redigiram o artigo. Ambos os autores aprovaram a versão final submetida.

## Agradecimentos.

Agradecemos aos professores Carmelo A. Juárez-Castelló, Cid M. M. Vianna e Roberto Rodríguez-Ibeas pelas significativas sugestões e revisão do texto.

## Declaração.

As opiniões expressas no manuscrito são de responsabilidade exclusiva dos autores e não refletem necessariamente a opinião ou política da RPSP/PAJPH ou da Organização Pan-Americana da Saúde (OPAS).
